# Effects of the therapy shift from cortisone acetate to modified-release hydrocortisone in a group of patients with adrenal insufficiency

**DOI:** 10.3389/fendo.2023.1093838

**Published:** 2023-01-24

**Authors:** Sofia Frigerio, Giulia Carosi, Emanuele Ferrante, Elisa Sala, Elisa Polledri, Silvia Fustinoni, Bruno Ambrosi, Iacopo Chiodini, Giovanna Mantovani, Valentina Morelli, Maura Arosio

**Affiliations:** ^1^ Endocrinology Unit, Fondazione Istituto di Ricovero e Cura a Carattere Scientifico (IRCSS) Ca’ Granda Ospedale Maggiore Policlinico, Milan, Italy; ^2^ Department of Clinical Sciences and Community Health, University of Milan, Milan, Italy; ^3^ Department of Experimental Medicine, Sapienza University of Rome, Rome, Italy; ^4^ Clinical Laboratory, Fondazione Istituto di Ricovero e Cura a Carattere Scientifico Istituto di Ricovero e Cura a Carattere Scientifico (IRCSS) Ca’ Granda Ospedale Maggiore Policlinico, Milan, Italy; ^5^ Istituto Clinico San Siro, Milan, Italy; ^6^ Unit of Endocrinology, Ospedale Niguarda-Ca' Granda, Milan, Italy; ^7^ Department of Medical Biotechnology and Translational Medicine, University of Milan, Milan, Italy; ^8^ Unit for Bone Metabolism Diseases and Diabetes, Istituto Auxologico Italiano Istituto di Ricovero e Cura a Carattere Scientifico (IRCCS), Milan, Italy

**Keywords:** adrenal insufficiency, salivary cortisol, Addison disease, hydrocortisone, modified-release hydrocortisone (MRH)

## Abstract

**Objective:**

Patients with adrenal insufficiency (AI) may be exposed to supraphysiological glucocorticoids levels during standard treatment with cortisone acetate (CA) or immediate-release hydrocortisone (IR-HC). Recent studies, predominantly including patients in IR-HC treatment, suggested that modified-release hydrocortisone (MRH) provide a more physiological cortisol rhythm, improving metabolic control and quality of life. Our primary aim was to assess clinical and biochemical modifications in patients shifted from CA to MRH.

**Design/Methods:**

We designed a retrospective longitudinal study, enrolling 45 AI patients (22 primary and 23 secondary AI) treated exclusively with CA thrice daily, shifted to MRH once daily; 29/45 patients concluded at least 18-months follow-up (MRH-group). We recruited 35 AI patients continuing CA as a control group (CA-group). Biochemical and clinical data, including metabolic parameters, bone quality, and symptoms of under- or overtreatment were collected. In 24 patients, a daily salivary cortisol curve (SCC) performed before and one month after shifting to MRH was compared to healthy subjects (HS).

**Results:**

No significant changes in glycometabolic and bone parameters were observed both in MRH and CA-groups during a median follow-up of 35 months. A more frequent decrease in blood pressure values (23.1% vs 2.8%, p=0.04) and improvement of under- or overtreatment symptoms were observed in MRH vs CA-group. The SCC showed a significant steroid overexposure in both CA and MRH-groups compared to HS [AUC (area under the curve) = 74.4 ± 38.1 nmol×hr/L and 94.6 ± 62.5 nmol×hr/L respectively, vs 44.1 ± 8.4 nmol×hr/L, p<0.01 for both comparisons], although SCC profile was more similar to HS in MRH-group.

**Conclusions:**

In our experience, patients shifted from CA to equivalent doses of MRH do not show significant glycometabolic modifications but blood pressure control and symptoms of over-or undertreatment may improve. The lack of amelioration in glucose metabolism and total cortisol daily exposure could suggest the need for a dose reduction when shifting from CA to MRH, due to their different pharmacokinetics.

## Introduction

Adrenal insufficiency (AI) is characterized by insufficient cortisol production; this can be caused by either primary adrenal failure (PAI - Addison’s disease) or inadequate secretion of ACTH from the pituitary gland (1, 2). In the absence of prompt replacement therapy, this condition is potentially lethal, thus treatment with glucocorticoids (GC) is essential. This treatment aims to reproduce the physiological circadian pattern of cortisol secretion (3) with higher levels in the morning that decrease during the day reaching the nadir at night, with adjustments during periods of stress (4, 5). However, classical replacement therapy with cortisone acetate (CA) or hydrocortisone (HC), administered with two or three daily doses, has been demonstrated to expose patients to supraphysiological cortisol levels (6). Side effects of cortisol excess are mainly represented by glycometabolic alterations, cardiovascular disease, hypertension, obesity, osteoporosis, risk of infections, and, consequently, a reduction of QoL and increased mortality (4, 6–8).

To overcome this limitation the once-daily modified-release hydrocortisone formulation (MRH, Plenadren^®^) has been developed: as demonstrated by pharmacokinetic studies and recently confirmed by the use of salivary cortisol, this drug allows to reach more physiological cortisol levels (9, 10). The meta-analysis by Bannon et al. (11) which collected data from nine studies including two randomized controlled trials (12, 13) demonstrated how MRH, compared to standard therapies, has a positive effect on body mass index, lipid profile, glycemic control, especially in diabetic subjects, and improves QoL, causing side effects comparable to those of classical treatments. Similar results were reported in a very recent study by Delle Cese et al. (14) that showed an improvement in metabolic control and QoL in a group of PAI switched from immediate-release hydrocortisone (IR-HC) to lower doses of MRH. However, only Guarnotta et al. (15) achieved a follow-up of 48 months; the other existing trials have a shorter duration. Few data are also available about the effects of MRH on bone quality (16). Therefore, further studies are needed to confirm the persistence of the benefits of the modified-release formulation in the medium to long term and to confirm the harmless effect of this therapy on bone health.

The primary aim of our study was to evaluate in our cohort of patients who shifted from CA (the most used substitutive glucocorticoid therapy in Italy) to MRH, the long-term effects on glucose metabolism, blood pressure, bone health, and subjective disease control. The secondary aim was to assess the effective total daily exposure to GCs, using salivary cortisol.

## Material and methods

### Subjects

**Figure 1:**
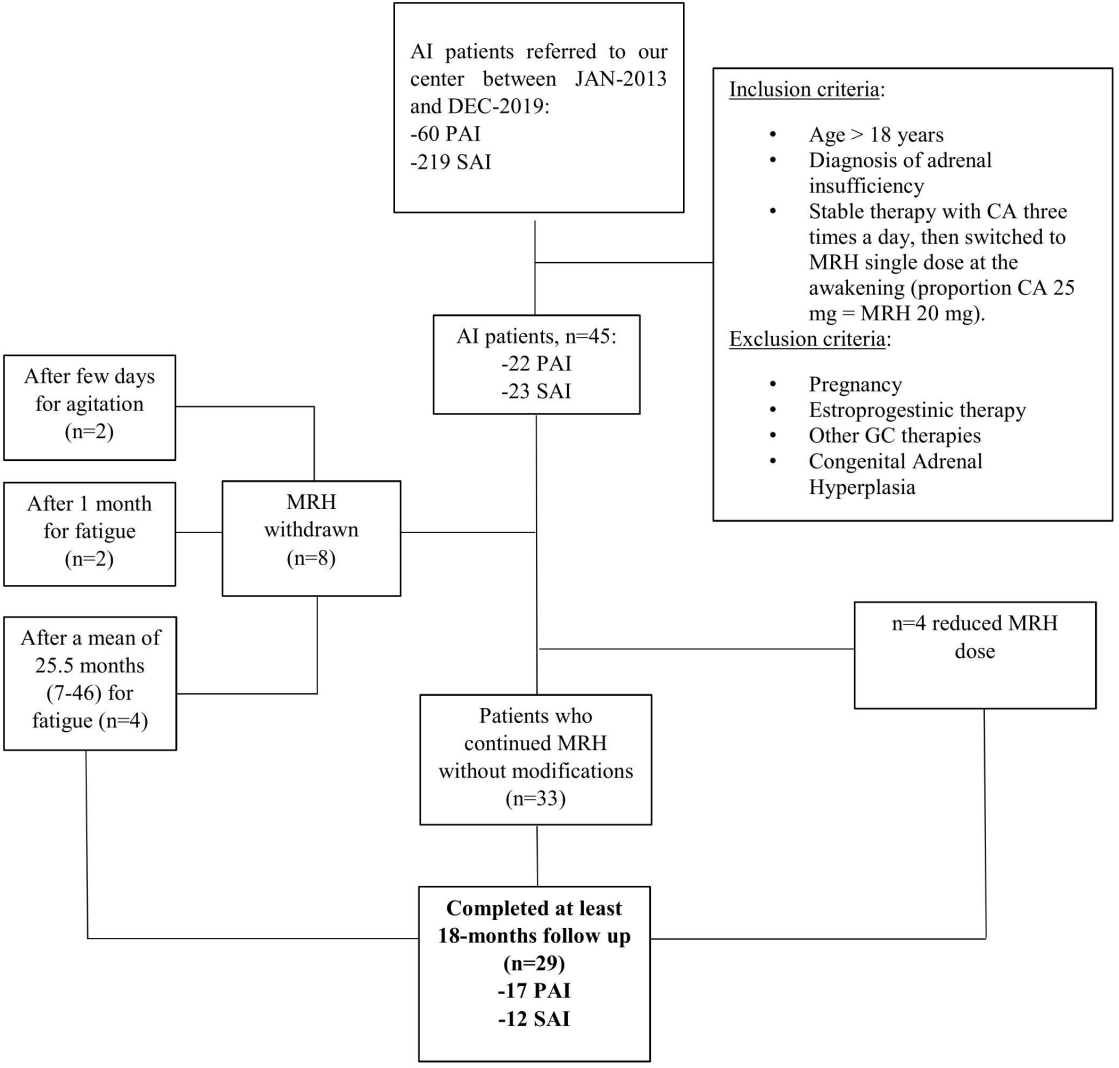
Study design and population. PAI, Primary Adrenal Insufficiency; SAI, Secondary Adrenal Insufficiency; CA, cortisone acetate; MRH, modified release hydrocortisone.

In this study we retrospectively evaluated data about 45 patients (30 females and 15 males, mean age 51.4 ± 14.3 years) referred to our tertiary care center for adrenal insufficiency between January 2013 and December 2019, respecting the inclusion and exclusion criteria shown in **Figure 1**. Among the study population, 22 patients were affected with primary adrenal insufficiency (PAI) and 23 patients with secondary adrenal insufficiency (SAI). All patients were previously taking CA three times a day (at the awakening, at 1:00 pm, and 5:00 pm) with the highest dose at the awakening, then shifted to an equivalent dose of MRH, considering that 25 mg of CA are equal to 20 mg of MRH. In particular, for MRH, available at the dose of 20 or 5 mg, not divisible, we used the 20/25 mgs formulation in most of the cases, with the exception of one patient taking 50 mg of CA which was shifted to 30 mg of MRH. The shifting was conducted because of inadequate disease control or to improve compliance. Eight out of 45 patients (17.7%) stopped therapy with MRH during the follow-up as shown in **Figure 1**. On the other hand, four patients had to reduce their dose (in 2 cases from 20 to 15 mg and in 2 cases from 25 to 20 mg) because of overtreatment symptoms (e.g. insomnia). All the remaining patients (33 out of 45) continued MRH without changing the dose.

Data about 29 patients (17 PAI and 12 SAI) that completed at least the 18-months have been analyzed and will be described in this study (MRH-Group). A group of 35 AI patients (19 PAI and 16 SAI), gender and age matched, who had continued classical therapy with cortisone acetate for at least 18 months after baseline evaluation, have been included as a control group (CA-Group).

In 28 PAI also treated with fludrocortisone, no modifications of mineralocorticoid substitutive therapy were reported during the follow-up (15/28, 12/28, and 1/28 were taking fludrocortisone 0.05, 0.1, and 0.025 mg/day, respectively).

All SAI patients affected with other concomitant pituitary deficiencies were receiving appropriate replacement therapy.

### Methods

Aim 1 - Long term outcomes: in the MRH-Group, we collected anthropometric data (weight, height, and BMI), glucose metabolism data, including fasting glucose and glycated hemoglobin (HbA1c), blood pressure values, and bone health assessment, including Dual-energy X-ray absorptiometry (DEXA) of the lumbar and femoral site, and spine X-rays for morphometric study. All these parameters were evaluated before and at least 18 months after the therapy shift. In the CA-Group, we collected the same data at the baseline (the first visit to our hospital) and at the last follow-up visit performed at least 18 months after. Blood samples for glucose metabolism were collected after GC morning dose administration. Improvement or worsening of glucose metabolism was defined according to the transition from a glycemic status category to another one [euglycemia, impaired fasting glucose (IFG) or diabetes mellitus (DM)] according to international criteria (17) and/or if hypoglycemic therapies were modified. We considered as significant the changes in the body weight of at least 5% from basal weight (18). Arterial hypertension (AH) was defined by the presence of systolic blood pressure (BP) values ≥140 mmHg and/or diastolic BP≥90 mmHg, or the use of antihypertensive drugs. We considered the improvement or worsening of BP according to the transition from an AH category to another one following the 2018 ESC/ESH Guidelines for the management of arterial hypertension (19) or as a modification in antihypertensive therapy. The presence of osteoporosis (OP) was defined by the presence of vertebral and/or lumbar T-score ≤ -2.5 standard deviation score (SDS) at the DEXA scan and morphometric vertebral fractures. We considered significant the improvement or worsening of bone health by evaluating the Least Significant Change (LS 2.8%, FN 5.9%) (20) in BMD (bone mineral density) values and/or onset of clinical or morphological fractures. We included in the analyses the spinal deformity index (SDI) which incorporates both the number and severity of vertebral fractures (21).

In addition, we assessed the subjective disease control by creating a score (SDC-score) which considered six typical symptoms of over or undertreatment (asthenia, myalgia, abdominal pain, muscle weakness, mental health related symptoms, insomnia): each symptom, if present, corresponded to one point so that a higher score was indicative of worse therapeutic control.

Aim 2 - Salivary cortisol daily curve: in 24 out of 45 subjects of the initial cohort of AI patients shifted to MRH, we analyzed the cortisol daily curve assessed with salivary cortisol samples to check therapy absorption and adequacy (time of collection: 8.30 am, 1 pm, 2.30 pm, 5 pm, 6.30 pm, and 10 pm). MRH and CA have been firstly administered in the morning at 7:00 am. The second dose of CA was administered at 1 pm (before salivary cortisol collection) and the third dose at 5 pm (before salivary cortisol collection). This assessment was performed at the baseline and one month after the therapy shift. We calculated the salivary cortisol area under the curve (SC-AUC) by trapezoidal integration as representative of the total daily exposure to cortisol and we compared it to age and gender-matched 24 HS’ values.

### Assays

All data about medical history, blood tests, and exams were collected from medical visit reports. Salivary samples had been collected by chewing a cylindrical cotton swab (Salivette^®^, Sarstedt, Nümbrecht, Germany). At least 3 hours before the collection, the subjects were told not to eat or brush their teeth to avoid contamination with blood, which contains vastly more cortisol than saliva. A liquid chromatography/tandem mass spectrometry (LC/MS/MS) method was used to measure salivary cortisol. The method had a precision, assessed as a percent coefficient of variation, of less than 10%, and accuracy between 93 and 107%, and a limit of quantification (LOQ) of 1 µg/L (22).

### Statistical analysis

Continuous and categorical variables have been compared between the two MRH/CA groups and in the same group (baseline vs. follow-up) respectively with t-test (or Mann-Whitney U test, if necessary) and test χ2 (or Fisher Exact test, if necessary). P values <0.05 have been considered statistically significant. Statistical analysis was performed by SPSS version 26.0 statistical package (SPSS Inc, Chicago, IL). If not differently specified, the results are expressed as mean ± SD or as % (n), or as median (interquartile range). The sample size was calculated on the expected amelioration of glycometabolic control: 17 subjects had to be enrolled to reach a power of 0.9 (type I error 0.01).

The Local Ethical Committee (Comitato Etico Milano Area 2) approved the protocol study.

## Results

Clinical and biochemical characteristics of the two groups at the baseline and follow-up are summarized in **Table 1**.

**Table 1:** Clinical and biochemical metabolic parameters at baseline vs long-term follow-up in MRH-Group and CA-Group.

	MRH-Group			CA-Group		
	Baseline (n=29)	Follow-up (n=29)	p	Baseline (n=35)	Follow-up (n=35)	p
Age, years	51.2 ± 13.0 (19-71)	54.4 ± 13.0 (22-74)	0.372	56.8 ± 14.9 (28-83)	59.9 ± 14.4 (33-87)	0.390
Gender	19 F, 10 M	19 F, 10 M	–	22 F, 13 M	22 F, 13 M	–
BMI, kg/m2	26.6 ± 5.4 (17.5-36.8)	26.0 ± 4.6 (17.9-35.7)	0.914	26.8 ± 5.0 (18.7-41.5)	26.9 ± 5.0 (19.1-37.8)	0.935
Weight, kg	70.7 ± 17.8 (40-110)	69.1 ± 15.9 (45-107)	0.714	71.1 ± 14.7 (45-120)	71.5 ± 14.1 (45-110)	0.918
Fasting glucose, mg/dl	89.6 ± 28.8 (69-158)	97.7 ± 28.8(69-194)	0.260	88.6 ± 17.3 (68-159)	95.8 ± 38.3 (68-296)	0.334
HbA1c, %	5.8 ± 0.9 (4.2-8.6)	6.2 ± 1.5 (4.9-10.6)	0.304	5.7 ± 0.8 (4.4-7.6)	5.8 ± 0.9 (4.6-9.4)	0.755
IFG % (n)	10.3 (3)	13.8 (4)	1	0 (0)	8.6 (3)	0.241
DM % (n)	20.7 (6)	20.7 (6)	1	14.3 (5)	14.3 (5)	1
SBP, mmHg	124.0 ± 16.9 (90-160)	118.6 ± 13.9 (95-150)	0.210	123.7 ± 16.2 (95-150)	124.7 ± 22.5 (80-160)	0.847
DBP, mmHg	75.9 ± 9.9 (60-100)	73.9 ± 6.6 (65-89)	0.387	76.7 ± 8.8 (60-90)	78.9 ± 13.5 (60-110)	0.440
AH % (n)	24.1 (7)	41.4 (12)	0.263	20.0 (7)	34.3 (12)	0.282
SDC-Score*	1 (1-2)	0 (0-1)	0.017	0 (0)	0 (0)	0.317
CA dose, mg/day	28.6 ± 6.6 (25-50)	–	–	28.7 ± 7.1 (18.6-50)	28.7 ± 7.3 (12.5-50)	1
CA therapy duration, months	107.6 ± 124.4 (8-456)	–	–	135.6 ± 171.3 (2-564)		–

BMI, Body Mass Index; IFG, impaired fasting glucose; DM, diabetes mellitus; SBP, systolic blood pressure; DBP, diastolic blood pressure; AH, arterial hypertension; SDC-Score, subjective disease control-score; CA, cortisone acetate; MRH-Group, patients that shifted CA therapy to MRH; CA-Group, CA patients who did not change therapy.

*Data expressed in median (interquartile range).

At the baseline evaluation, patients of MRH-Group and CA-Group were comparable regarding the dose of CA, duration of therapy with CA, age, gender, weight, BMI, prevalence of arterial hypertension, IFG, DM, OP, and frailty fractures, whereas SDC-score was significantly lower in CA-Group than in patients of MRH-Group [median score = 0 vs 1 (1-2), respectively, p<0.01], as expected, considering that symptoms of under/over treatment were considered for shifting to MRH.

Aim 1. Long-term outcomes: in the MRH-Group the mean MRH therapy duration was 38.1 ± 18.1 months, median 35 months (18-72), with a mean MRH dose of 21.8 and 20.9 mg/die at the beginning and at end of follow-up respectively (p=0.1). The CA-Group had a comparable mean follow-up duration [37.2 ± 16.3 months, median 35 months (18-72)], and a mean unchanged CA dose of 28.75 mg/die.

**Figure 2:**
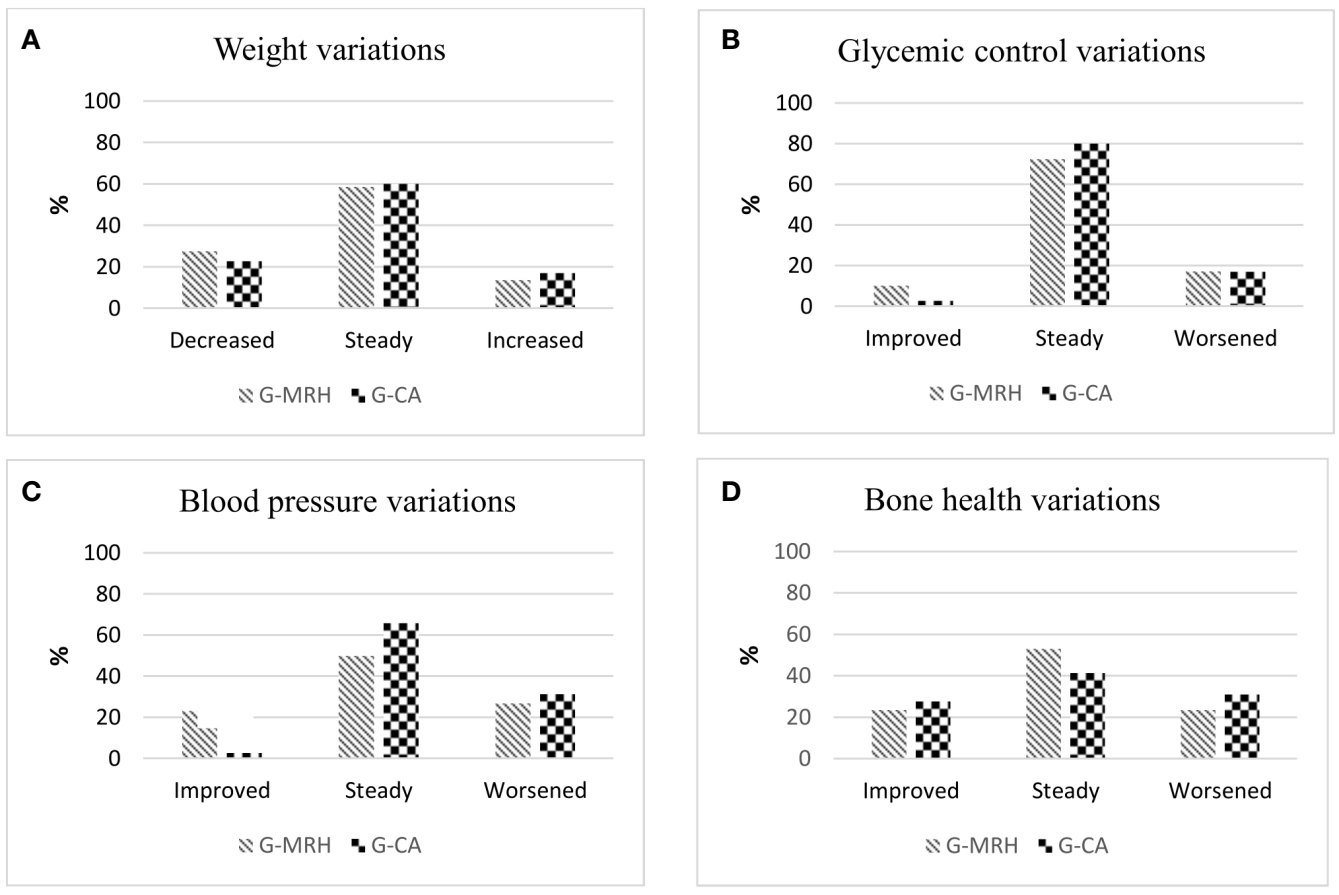
Rate of patients who displayed a modification or stability in weight **(A)** glycemic control **(B)**, blood pressure **(C)** and bone health **(D)** in the Group of patients that shifted cortisone acetate therapy to modifed-release hydrocortisone (G-MRH) and in the Group of patients who did not change cortisone acetate therapy (G-CA). We considered as significant the changes in the body weight of at least 5% from basal weight (18). Glycemic control was considered improved/worsened according to modification in concomitant hypoglycemic therapies or glycemic status (euglycemia, IFG, or DM) (17). BP control was considered improved/ worsened in case of variations in BP values or antihypertensive therapy (19). We considered significant the improvement or worsening of bone health by evaluating the Least Significant Change (20) in BMD values and/or onset of clinical or morphological fractures. IFG, impaired fasting glucose; DM, diabetes mellitus; BP, blood pressure; BMD, bone mineral density.

*Blood pressure and glucose metabolism*. Percentages of patients stable, improved, or worsened are shown in **Figure 2**. Concerning blood pressure, 5 patients with normal BP at baseline of both MRH-Group and CA-Group became hypertensive during follow-up. Even though AH prevalence remained comparable (41.4% MRH vs. 34.3% CA-Group, p=0.611), blood pressure control improved more frequently in MRH than CA-Group patients considering the reduction of antihypertensive treatment or shifting to another AH class (improvement in 23.1% vs 2.8% patients, p=0.04) (**Figure 2**). No statistically significant changes in glucose metabolism were observed over time in both groups. IFG and DM prevalence remained comparable at follow-up.

*Bone health.* In **Table 2** are summarized data of 17/29 subjects in MRH-Group and 27/35 subjects in CA-Group, whose bone health had been evaluated at the baseline. During follow-up, two patients of both groups became osteoporotic, consequently, the prevalence of OP at follow-up remained still comparable. As shown in **Figure 2D**, no significant variations in bone health assessment were observed comparing the two groups. Moreover, in MRH-Group no patients experienced clinical or morphological fractures during follow-up, while three subjects of CA-Group presented a new vertebral fracture at X-rays follow-up (one patient with a previous vertebral fracture and two naïve patients) (0 vs. 13%, p=0.536). No significant difference in SDI score emerged among groups (**Table 2**).

**Table 2:** Bone health assessment at baseline vs. long-term, MRH-Group and CA-Group.

	MRH-G			CA-G		
	Baseline (n=17)	Follow-up (n=17)	p	Baseline (n=27)	Follow-up (n=27)	P
OP % (n)	35.3 (6)	47.1 (8)	0.728	48.1 (13)	55.6 (15)	0.786
VFX % (n) *	40.0 (4)	40.0 (4)	1	17.4 (4)	26.1 (6)	0.722
SDI°*	0 (0-1.5)	0 (0-1.5)	1	0 (0)	0 (0-1)	0.102
LS Z-sc, SDS	-0.5 ± 1.4 (-2.2 – 2.2)	-0.5 ± 1.4 (-2.7 – 2.2)	0.502	-0.2 ± 1.7 (-3.7 – 3.8)	-0.1 ± 1.8 (-3.1 – 4.2)	0.259
FN Z-sc, SDS	-0.5 ± 1.1 (-2.5 – 1.7)	-0.5 ± 1.1 (-2.1 – 1.6)	0.400	-0.3 ± 0.9 (-1.9 – 1.4)	-0.3 ± 0.9 (-2.0 – 1.8)	0.517
FT Z-sc, SDS	-0.3 ± 1.3 (-2.5 – 2.5)	-0.3 ± 1.2 (-2.2 – 2.3)	0.718	-0.1 ± 1.1 (-2.0 – 2.8)	-0.1 ± 1.1 (-1.8 – 2.4)	0.422

MRH-G, MRH-Group; CA-G, CA-Group; OP, osteoporosis; VFX, prevalence of patients with vertebral fractures; SDI, Spinal Deformity Index; LS, lumbar spine; FN, femoral neck; FT, femoral total; Z-sc, Z-score; SDS, standard deviation score.

*Data about vertebral fractures in 10 subjects in G-MRH and 23 subjects in G-CA.

°Data expressed in median (interquartile range).

*Subjective disease control*. The SDC-score used to evaluate the presence of symptoms typical of bad subjective compensation showed a significant improvement in patients treated with MRH over time [median 1 (1-2) vs. 0 (0-1), p =0.017, baseline and follow-up, respectively]. In CA-Group, SDC-score remained stable during follow-up [median 0 (0) vs. 0 (0), *p* = 0.317]. No hypoadrenal crises were reported during the follow-up period.

**Figure 3:**
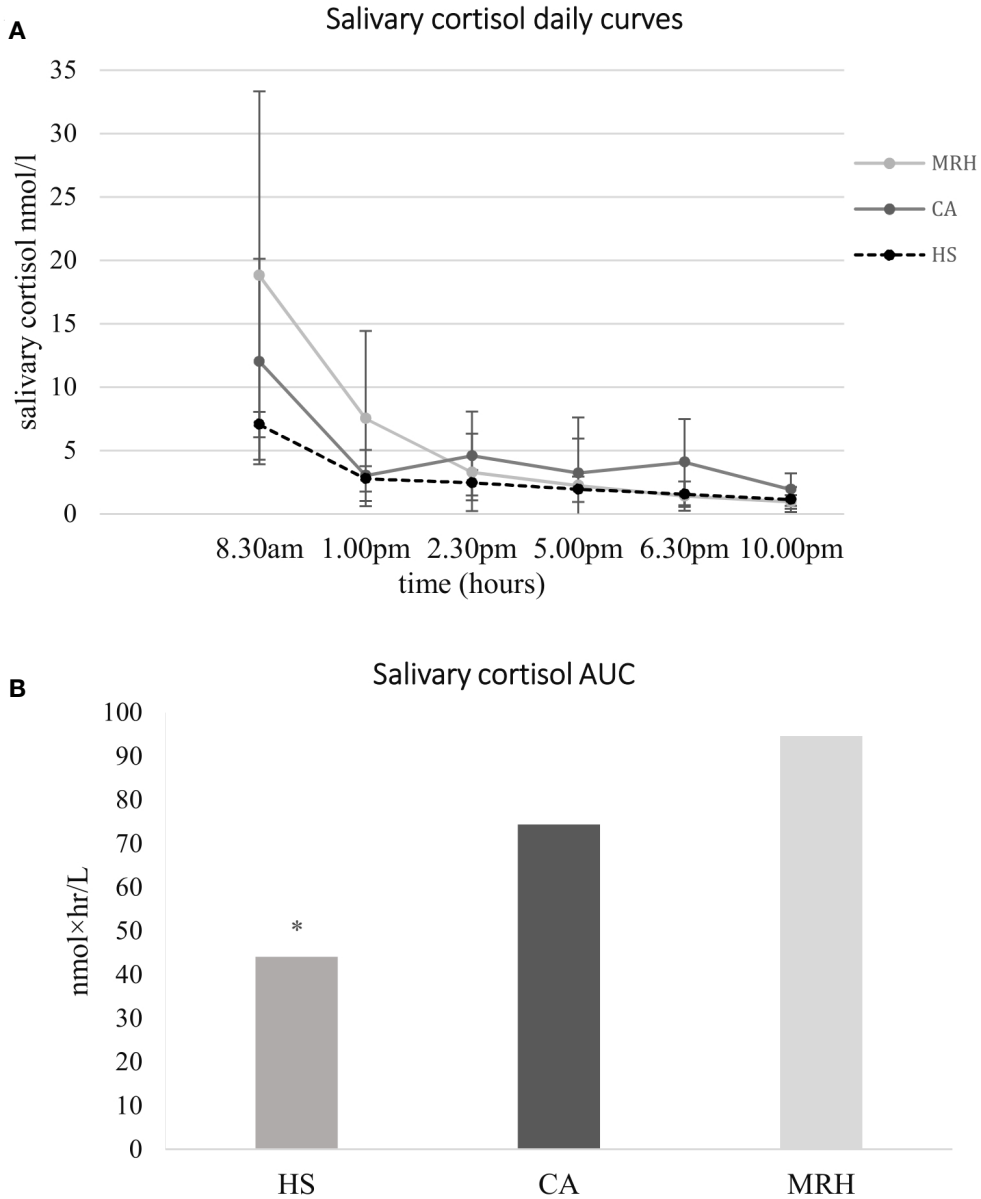
Salivary cortisol daily curves **(A)** and cortisol Area Under the Curve **(B)** at baseline (patients in therapy with cortisone acetate, CA) and 1 month after therapy modification to modifed-release hydrocortisone (MRH), both compared to salivary cortisol levels collected in healthy subjects (HS). MRH (light grey line); CA, (dark grey line); HS (dotted line); AUC, Area Under the Curve; Salivary cortisol AUC has been calculated by trapezoidal integration as representative of the total daily exposure to cortisol. * p = 0.009 vs both CA and MRH.

Aim 2. Salivary cortisol daily curve: data about salivary cortisol daily curves collected at baseline and one month after therapy shift are summarized in **Figure 3**, both compared to salivary cortisol levels collected in HS. The SA-AUC showed a significant overexposure of subjects treated both with CA and with MRH compared to HS (74.4 ± 38.1 nmol×hr/L vs. 44.1 ± 8.4 nmol×hr/L, *p* = 0.01; 94.6 ± 62.5 nmol×hr/L vs. 44.1 ± 8.4 nmol×hr/L, respectively, *p* = 0.009). SA-AUC of CA-group tended to be lower than MRH one (*p* = 0.051), in particular, in the former hours of the day, cortisol exposure was significantly higher with MRH versus CA (at 8.30 am 18.8 ± 14.5 nmol/l vs. 12.0 ± 8.1 nmol/l, *p* = 0.004; at 1 am 7.5 ± 6.9 nmol/l vs 3.0 ± 2.0 nmol/l, respectively *p* = 0.002). During the day, as it is shown in **Figure 3**, the difference between CA and MRH decreased, then reversed in the second half of the day (at 6.30 pm 4.1± 3.4 nmol/l vs. 1.4 ± 1.1 nmol/l, *p* = 0.001; at 10 pm 1.9 ± 1.3 nmol/l vs. 0.9 ± 0.5 nmol/l, *p* = 0.000, respectively).

Regarding the cortisol profile during the day, patients treated with CA showed a cortisol exposure significantly higher than HS in correspondence with three peaks, concomitant with the three daily drug administrations: (CA vs. controls) at 8.30 am 12.0 ± 8.0 nmol/l vs. 7.1 ± 2.4 nmol/l, *p*=0.046; at 2.30 pm 4.6 ± 3.5 nmol/l vs. 2.4 ± 0.5 nmol/l, *p* = 0.045, at 6.30 pm 4.1 ± 3.4 nmol/l vs. 1.6 ± 0.3 nmol/l, *p*=0.015. One month after shifting to MRH, cortisol exposure showed a cortisol profile more similar to HS, higher in the morning which decreased during the afternoon, but with significant differences in the first half of the day: at 8.30 am 18.8 ± 14.5 nmol/l vs. 7.1 ± 2.4 nmol/l, *p* = 0.009; at 1 pm 7.5 ± 6.9 nmol/l vs. 2.8 ± 0.8 nmol/l, p = 0.025 (MRH vs. HS).

## Discussion

Our study described a cohort of hypoadrenal patients who shifted from classical replacement therapy with cortisone acetate to modified-release hydrocortisone. MRH was well tolerated and continued in most patients without modifications. Data on glycometabolic, cardiovascular, and bone health assessment after shifting to MRH were compared to patients continuing CA. Over a median follow-up of 35 months, we did not observe any significant modification in all the examined parameters in both groups except for arterial hypertension control and SDC-score, which improved more frequently in patients who shifted to MRH. The analyses of the salivary cortisol curve showed that daily cortisol profile with MRH treatment was more similar to healthy subjects than CA-group. However, in both MRH and CA groups, the overall cortisol daily exposure was higher than controls, especially in the first part of the day with MRH and in the second part with the CA.

In hypoadrenal patients, GCs replacement aims to mimic the physiological trend of daily cortisol, avoiding adrenal crisis and possible consequences of higher GCs doses exposure (1–4). In the last years, MRH has been developed to reach a more physiological range of cortisol levels (9). To the best of our knowledge, this is the first study including patients taking CA as the unique GC formulation, that shifted to MRH. It is well known that, due to its formulation and kinetics, MRH is immediately active after absorption, differently from CA which requires the hepatic passage for its activation. Thus, it is possible to hypothesize that patients who shifted from CA to MRH could need a lower dose of the drug, to avoid a hypothetical overtreatment. In our study patients taking CA were shifted to an equivalent dose of MRH, considering that 25 mg of CA are equal to 20 mg of MRH. In particular, for MRH, available at the dose of 20 or 5 mg, not divisible, we used the 20/25 mgs formulation in most of the cases, with the exception of one patient taking 50 mg of CA which was shifted to MRH 30 mg. This could explain why in our cohort of patients the positive MRH effects on glucose metabolism, described in other studies, were not observed. Indeed, available studies (11–13) demonstrating how MRH, compared to classical replacement therapies, exerted positive effects on glycometabolic parameters, included almost exclusively patients in IR-HC. Especially in patients taking CA doses higher than 25 mg the use of MRH, with fixed dose of 20 mg available, could help to reduce treatment, without the occurrence of withdrawal symptoms.

Among the longitudinal studies focusing on the glyco-lipidic metabolic effects of MRH, only the retrospective study by Guarnotta et al. (15) achieved a follow-up of 48 months. In a similar way, our study assessed different parameters on a long follow-up (18-72 months with MRH), and then made a comparison with a CA control group. As opposed to this study, we did not observe any significant modification of anthropometric parameters and glycemic control after the therapy shift. In particular, the prevalence of diabetes did not change, both in MRH and CA groups, even though glucose metabolism tended to improve more in patients on MRH, without reaching statistical significance. At variance with the study of Guarnotta et al. (15) in our cohort the percentage of subjects with glycemic impairment and the mean follow-up duration were lower, possibly limiting the detectable positive metabolic effect of MRH. On the other way, our results are similar to those reported in the prospective study by Ceccato et al. (10).

We also evaluated blood pressure control as a marker of cardiovascular risk and sign of overtreatment. In addition to literature data, despite five patients of both groups becoming hypertensive during follow-up, we found that patients of MRH-Group reached a better blood pressure control than patients in CA, with no adjustment of mineralocorticoid substitutive therapy. This result could suggest a minor impact of MRH than CA on the cardiovascular system, and, considering that the prevalence of hypertension in our cohort of patients is not negligible, this result may be of clinical importance. In a previous study, a modification in blood pressure, especially the diastolic blood pressure, in patients with AI had been reported, after the reduction of median 5 mg HC glucocorticoid dose (23). In our study, we can hypothesize that the improvement in BP control may be not related to a different dose of GCs but to the different GCs exposure during the day provided by MRH. Indeed, in the MRH-Group the mean MRH therapy dose was not statistically different comparing the beginning and the end of follow-up.

Regarding the risk of osteoporosis, to date, only the study by Frara et al. (16) evaluated the effects of MRH on bone mass in a small group of patients with secondary AI. In that study seems that a more physiological trend of daily cortisol could have a positive effect on bone metabolism, leading to increased BMD values at the lumbar spine and femoral neck after 24 months of treatment with MRH. In our study, no significant effects on BMD emerged in patients treated with MRH, also compared to patients continuing CA. Our study is the first that assessed the incidence of vertebral fractures. We observed that morphological vertebral fractures occurred during follow-up only in the group continuing CA, even without reaching statistical significance, possibly due to the low number of cases.

The difficulty in achieving adequate replacement therapy mainly derives from the poor reliability of the biochemical parameters available (urinary free cortisol, ACTH, serum cortisol), making particularly useful the careful evaluation of the clinical parameters, symptoms, and quality of life reported by the patient. Salivary cortisol, since it is easy to perform and represents a non-invasive method to determine free circulating cortisol levels, has been proposed as a useful parameter in the assessment of drug exposure in these patients (10). Although clinical and biochemical scores have been proposed, to date there is no gold standard for evaluating the therapeutic adequacy of these patients. The appropriate management of symptoms referable to over-or under-treatment is still an issue of discussion today. Some Authors recently pointed out a probable overtreatment using GC doses suggested by international guidelines (24). Therefore, it is necessary to conduct further studies that can allow standardizing the method of assessing disease control in patients with AI and related therapy adjustments. In our experience, the cortisol daily exposure in patients treated both with CA and with MRH was significantly higher than in healthy subjects. Anyway, it is important to consider that in healthy subjects cortisol levels gradually increase in the second half of the night, reaching their peak in the early morning (10). As we collected data from the first sample of salivary cortisol at 08.30 a.m. in both patients and HS, probably in the latter we missed their highest cortisol level. For this reason, the SC-AUC result in controls may be underestimated. The total daily exposure to GCs with MRH was even higher than CA, differently from literature data, almost reaching statistical significance. However, as stated before, this is the first study reporting SC levels in patients taking exclusively CA as classic replacement therapy. All previous studies have considered patients treated only with IR-HC or subgroups of patients treated with either IR-HC or with CA (3, 11–16). In a study by Feek et al. (25), comparing patients treated with IR-HC 20 mg to the ones on a corresponding replacement dose of CA (25 mg), IR-HC was associated with higher mean cortisol and lower mean ACTH levels. These results could be explained with the different pharmacokinetics of the two drugs. A larger salivary bioavailability of MRH compared to CA could also be hypothesized. Moreover, as well known, the cortisol trend during the day is very different in patients treated with classical replacement therapy and with MRH: while CA shows different peaks following daily administrations, MRH provides a curve that is more similar to the physiological one (10). This could be the reason why some patients, after the therapy switch, complained of the appearance of fatigue, the main reason for MRH withdrawal. This is not surprising if we consider that patients treated with CA are accustomed to being exposed to supra-physiological cortisol levels in the second half of the day. Despite this, we observed a significant reduction in symptoms of bad subjective disease compensation. The score we used (which assessed the presence of asthenia, myalgia, abdominal pain, muscle weakness, mental health related symptoms, and insomnia), significantly decreased from the baseline to follow-up. As expected, the group treated with CA showed steadily lower score values compared to MRH-group, because they were subjectively satisfied with their classical replacement therapy and a change in GC formulation was not indicated.

Our study suffers from many limitations and its retrospective nature is the main one. Moreover, the number of patients included is quite low, but similar to other studies about this topic (e.g. Guarnotta 2018 et al. 16). Nevertheless, the study follow-up is longer than most of the available studies concerning replacement therapy with MRH in real life and we included a control group for secondary analysis. Other limitations of the study are the variability in the follow-up duration among patients, and that data about some variables, such as vertebral fractures, are partial. Moreover, we are aware that the score we used to evaluate the subjective disease control, is not validated and we do not propose it as a substitute for internationally applied questionnaires, however, we decided to explore the main subjective health status symptoms (26) using a simple score. Quality of life questionnaires specifically tailored for AI patients often appear hardly evaluable in clinical practice. Regarding metabolic parameters, we only assessed glycemic control, and no data about lipids were analyzed. However, this is the first study comparing CA with MRH, suggesting the need for a tailored therapy shift due to the different pharmacokinetics of the two drugs.

In conclusion, in our experience, we found a globally positive role of MRH in AI treatment, especially in blood pressure control and subjective disease control. The lack of amelioration in glucose metabolism and total cortisol daily exposure could suggest the need for a dose reduction when shifting from CA to MRH. More studies, with a longer follow-up and larger cohorts, are needed to confirm possible positive effects on bone health.

## Data availability statement

The raw data supporting the conclusions of this article will be made available by the authors, without undue reservation.

## Ethics statement

The studies involving human participants were reviewed and approved by The Local Ethical Committee -Comitato Etico Milano Area 2. The patients/participants provided their written informed consent to participate in this study.

## Author contributionss

SoF and GC: data acquisition, analysis, and drafting of the manuscript. VM: study design, data acquisition, analysis and interpretation of the data. SiF, EP: data acquisition and analysis. EF, ES, BA: data acquisition. IC, GM, MA: critical revision of the manuscript for important intellectual content, and study supervision. All authors contributed to the article and approved the submitted version.
